# Supplementary figures and images for: Secreted antigen A peptidoglycan hydrolase is essential for Enterococcus faecium cell separation and priming of immune checkpoint inhibitor therapy
Source: eLife. 2024 Jun 10;13:RP95297. doi: 10.7554/eLife.95297 (PMC11164530; doi:10.7554/eLife.95297)

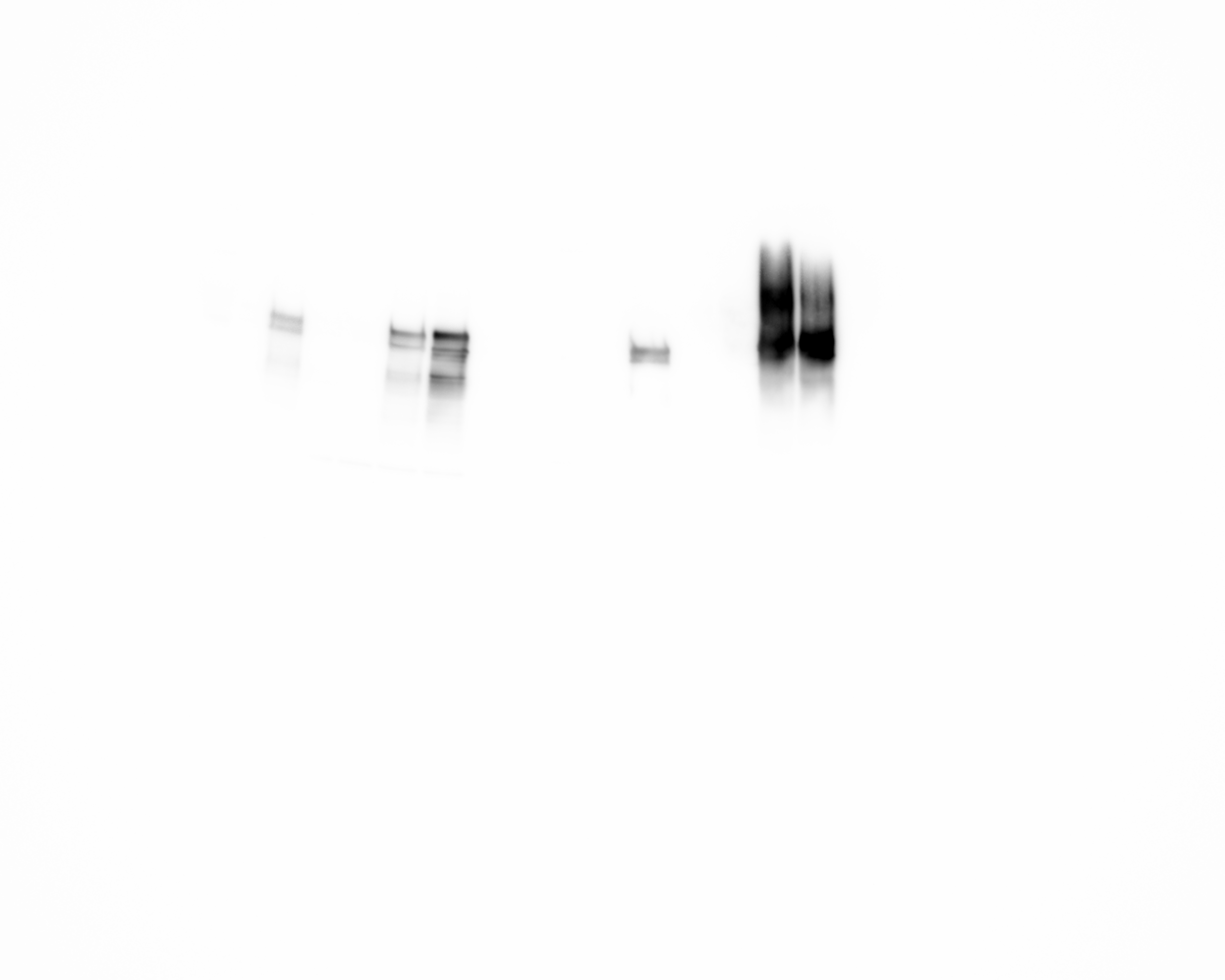

Supplement: Figure 1—source data 2. [file elife-95297-fig1-data2.zip › Figure 1 - source data 2/Fig 1b anti-SagA.tif]

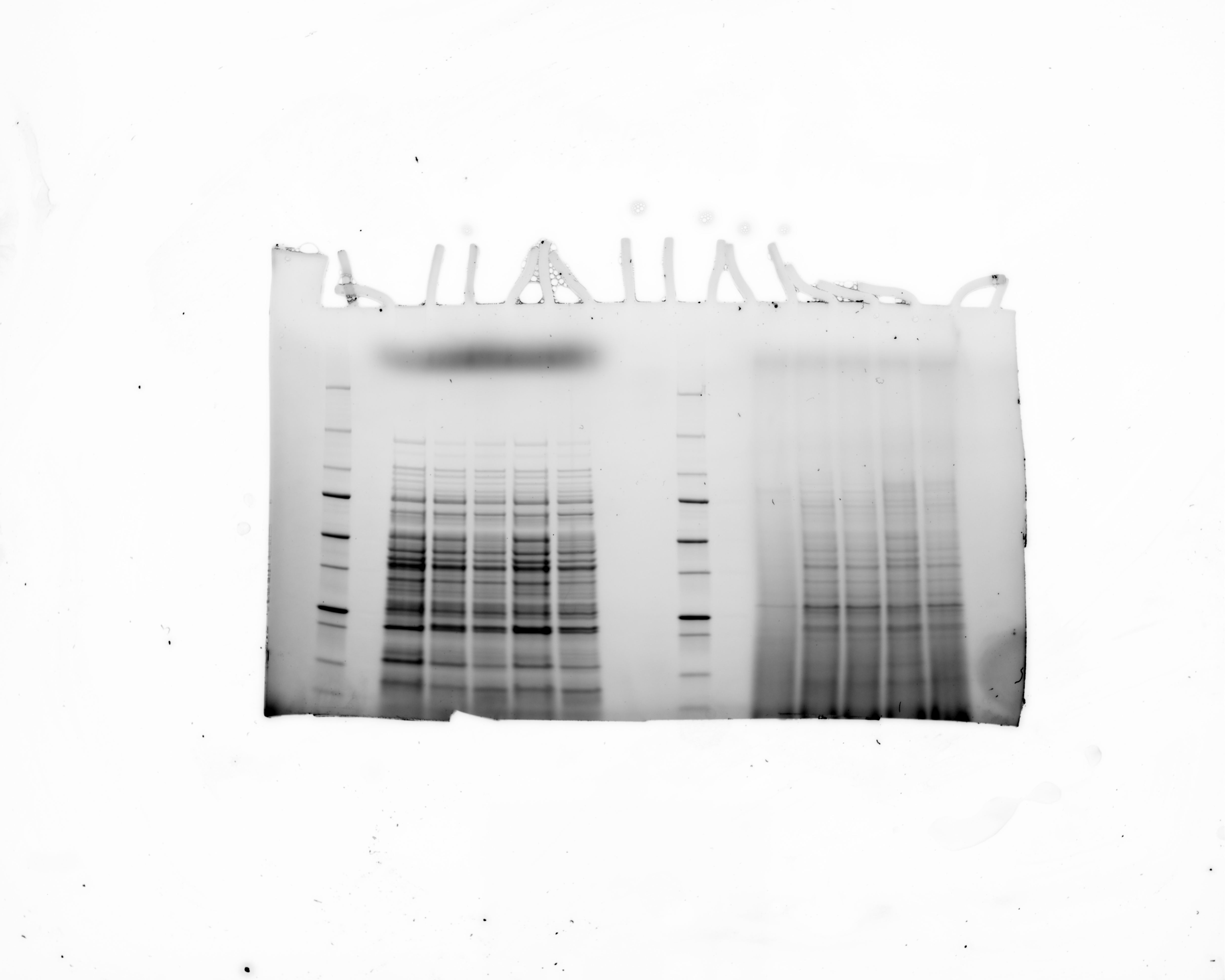

Supplement: Figure 1—source data 2. [file elife-95297-fig1-data2.zip › Figure 1 - source data 2/Fig 1b Total protein.tif]

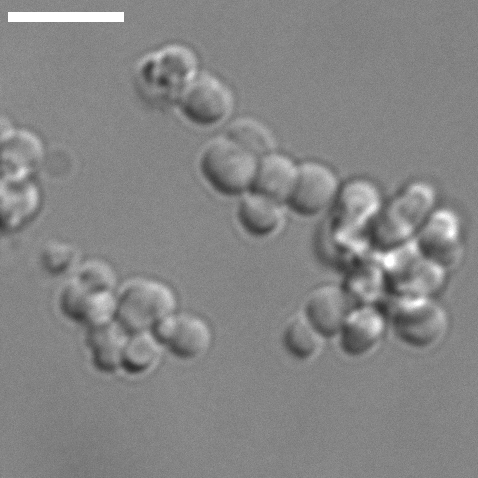

Supplement: Figure 1—source data 3. [file elife-95297-fig1-data3.zip › Figure 1 - source data 3/sagA KO.tif]

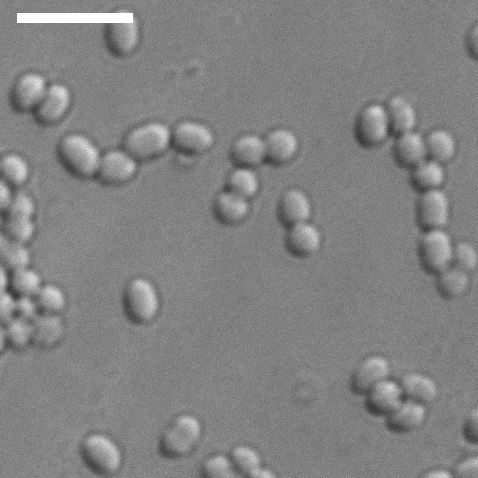

Supplement: Figure 1—source data 3. [file elife-95297-fig1-data3.zip › Figure 1 - source data 3/sagA-psagA.tif]

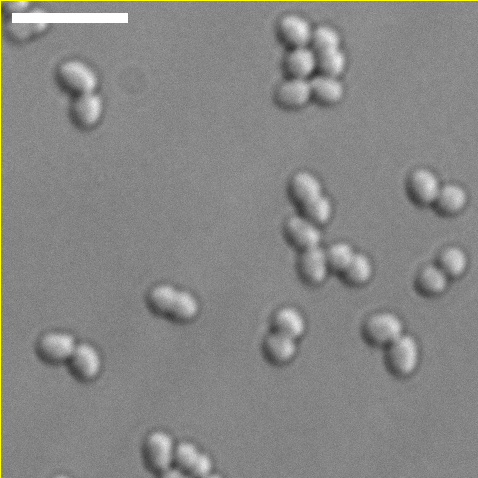

Supplement: Figure 1—source data 3. [file elife-95297-fig1-data3.zip › Figure 1 - source data 3/WT.tif]
