# Supplementary material for: Secreted antigen A peptidoglycan hydrolase is essential for Enterococcus faecium cell separation and priming of immune checkpoint inhibitor therapy
Source: eLife. 2024 Jun 10;13:RP95297. doi: 10.7554/eLife.95297 (PMC11164530; doi:10.7554/eLife.95297)
Supplement: Supplementary file 1. [file elife-95297-supp1.docx]

**Supplementary File 1. Mutations detected in ΔsagA E. faecium Com15 strain.**

| Genomic locus | BioCyc annotation | Type of mutation | Notes |
| --- | --- | --- | --- |
| EFWG_00994 | Mannosyl-glycoprotein endo-beta-n-acetylglucosaminidase | missense (L200F) | L200 is a conserved residue among GH73 family proteins |
| EFWG_01683 | 50S ribosomal protein L4 | missense (E16K) |  |
| EFWG_02413 | aquaporin family protein | missense (A45T) |  |
| EFWG_02113 | folate family ECF transporter S component | promoter  (-10 site) | promoter identified with BPROM |
| EFWG_01149 | LacI family transcriptional regulator | silent (L270L) |  |
| between EFWG_01661 and EFWG_01662 | N/A | intergenic |  |
| between EFWG_02410 and EFWG_02411 | N/A | intergenic |  |
| upstream of EFWG_02718 | N/A | intergenic |  |
| immediately downstream of EFWG_04048 | N/A | intergenic |  |
| EFWG_02083 | collagen-binding protein | insertion/ duplication | There is an apparent duplication of a 78bp fragment, however it is adjacent to a stretch of undefined sequence. Thus, this mutation may not truly be present, and may just be a result of incomplete sequencing. |
| EFWG_11565 | pseudogene | 3x silent (A451A, A453A, A457A) and insertion/  frameshift | EFWG_RS11565 is annotated in BioCyc as a pseudogene because of a premature stop codon that splits the gene into two ORFs. The frameshift mutation in Δ*sagA* returns the gene to a single ORF. |
