# Supplementary material for: Secreted antigen A peptidoglycan hydrolase is essential for Enterococcus faecium cell separation and priming of immune checkpoint inhibitor therapy
Source: eLife. 2024 Jun 10;13:RP95297. doi: 10.7554/eLife.95297 (PMC11164530; doi:10.7554/eLife.95297)
Supplement: Supplementary file 2. [file elife-95297-supp2.docx]

**Supplementary File 2. Summary of MIC determinations via antibiotic test strips for E. faecium WT, ΔsagA and ΔsagA/ psagA (Extended Data Fig. 2a).**

| Antibiotic | WT MIC (μg/ mL) | Δ*sagA*  MIC (μg/ mL) | Δ*sagA*/ p*sagA*  MIC (μg/ mL) | Change |
| --- | --- | --- | --- | --- |
| Tigecycline | ~ 0.125 | ~ 0.064 | ~ 0.125 | Yes |
| Telavancin | ~ 0.094 | ~ 0.094 | ~ 0.094 | No |
| Ceftriaxone | ~ 3 | ~ 0.75 | ~ 3 | Yes |
| Imipenem | ~ 1.5 | ~ 0.19 | ~ 0.50 | Yes |
| Fosfomycin | ~ 32 | ~ 8 | ~ 32 | Yes |
| Daptomycin | ~ 6 | ~ 2 | ~ 6 | Yes |
| Vancomycin | ~ 2 | ~ 2 | ~ 2 | No |
| Linezolid | ~ 6 | ~ 2 | ~ 6 | Yes |

MIC, minimum inhibitory concentration.
