# Supplementary material for: Secreted antigen A peptidoglycan hydrolase is essential for Enterococcus faecium cell separation and priming of immune checkpoint inhibitor therapy
Source: eLife. 2024 Jun 10;13:RP95297. doi: 10.7554/eLife.95297 (PMC11164530; doi:10.7554/eLife.95297)
Supplement: Supplementary file 3. [file elife-95297-supp3.docx]

**Supplementary File 3. E. faecium strains used in this study.**

| Strain | Description | Source |
| --- | --- | --- |
| *E. faecium* | *E. faecium* Com15 | lab stock |
| VC109 | Com15 harboring the pRecT plasmid | Chen V et al.^20^ |
| SK057 | Δ*sagA* | this work |
| SK080 | Δ*sagA*/ empty vector | this work |
| SK081 | Δ*sagA*/ p*sagA^C431A^* | this work |
| SK106 | *E. faecium* wt/ empty vector | this work |
| SK109 | Δ*sagA*/ p*sagA(v2)* | this work |
