# Supplementary material for: Secreted antigen A peptidoglycan hydrolase is essential for Enterococcus faecium cell separation and priming of immune checkpoint inhibitor therapy
Source: eLife. 2024 Jun 10;13:RP95297. doi: 10.7554/eLife.95297 (PMC11164530; doi:10.7554/eLife.95297)
Supplement: Supplementary file 4. [file elife-95297-supp4.docx]

**Supplementary File 4. Plasmids used in this study.**

| Plasmid | Description | Source |
| --- | --- | --- |
| pET21a | backbone used for assembling deletion constructs | lab stock |
| pAM401 | backbone for plasmids to be transformed into *E. faecium* | lab stock |
| pRecT | recombinase-harboring plasmid used to facilitate *E. faecium* deletions | Chen V et al.^20^ |
| p*sagA* | pAM401-based plasmid that contains *sagA* under the control of its native promoter | Espinosa J et al.^21^ |
| pSK060 | modified p*sagA* that swaps a cm^R^ cassette for an erm^R^ cassette (referred to in the main text, and in the plasmids below as “p*sagA*”) | this work |
| pSK069 | pAM401 with erm^R^ empty vector | this work |
| pSK071 | p*sagA^C443A^* | this work |
| pSK104 | p*sagA(v2)* (referred to as “p*sagA*” in the main text) | this work |
