# Supplementary material for: Secreted antigen A peptidoglycan hydrolase is essential for Enterococcus faecium cell separation and priming of immune checkpoint inhibitor therapy
Source: eLife. 2024 Jun 10;13:RP95297. doi: 10.7554/eLife.95297 (PMC11164530; doi:10.7554/eLife.95297)
Supplement: Supplementary file 5. [file elife-95297-supp5.docx]

**Supplementary File 5. Primers used in this study for generating complementation plasmids and empty vector.**

| Deletion | PCR Fragment Generated | Primer Name | Sequence (5’ to 3’) |
| --- | --- | --- | --- |
| SagA | homology arm 1 | oSK025 | TTTTGTTTAACTTTAAGAAGGAGATATACAAACTCCATGCATTAGTGGTGAC |
|  |  | oSK026 | ATCTATCTTGAGTTTCAATCTATTTTTTTTTCTTCATTCCTCCGACTGGC |
|  | cm^R^ cassette | oSK008 | TAAATTTAACGATCACTCAAAAAATTATAAAAGCCAGTC |
|  |  | oSK009 | AAAAAAAATAGATTGAAACTCAAGATAGATATGTTATTG |
|  | homology arm 2 | oSK027 | TTTATAATTTTTTGAGTGATCGTTAAATTTAGAATGATTAATCAAAAAGAGTTAGTCCAG |
|  |  | oSK028 | GCCGGATCTCAGTGGTGGTGGTGGTGGTGCGTAAGGAAAAAATGGAATGGCAG |
|  | complete deletion construct | oSK041 | AACTCCATGCATTAGTGGTGAC |
|  |  | oSK042 | GTAAGGAAAAAATGGAATGGCAG |
|  | verification of deletion | oSK368 | CTTTCTAACTATGAACTTCATGGC |
|  |  | oSK369 | TCCAGTACGGGTATCTTC |

| Purpose | Fragment | Primer Name | Sequence (5’ to 3’) |
| --- | --- | --- | --- |
| generating pSK060 by swapping cm^R^ for erm^R^ in p*sagA* using two-fragment Gibson assembly | p*sagA* backbone | oSK406 | TTACTTCCAAAACCTAAATTCACGTTG |
|  |  | oSK407 | TAAATTATTTAATAAGTAATTTTTAGATTTTGAAAGTGAATTTAATTTTATACACGTAAG |
|  | erm^R^ cassette | oSK408 | GATTTTTGGCAACGTGAATTTAGGTTTTGGAAGTAACATGTTCATATTTATCAGAGCTCG |
|  |  | oSK409 | TTCAAAATCTAAAAATTACTTATTAAATAATTTATAGCTATTGAAAAGAGATAAGAATTG |
| generating pSK069 by removing *sagA* from p*sagA* using Q5-SDM (NEB) | linearized pSK069 plasmid | oSK475 | GTCGACCGATGCCCTTGAGAGC |
|  |  | oSK476 | AGGATCCACAGGACGGGTGTG |
| generating pSK104 using two-fragment Gibson assembly | pSK060 backbone | oSK562 | TATGTTTGGGGCGGTAAAGATC |
|  |  | oSK563 | AGTGTAGGCCCTAAGAACCTTG |
|  | alternate *sagA* sequence | oSK564 | TTTTATTACAAGGTTCTTAGGGCCTACACT |
|  |  | oSK565 | TCCACTTGGATCTTTACCGCCCCAAACATA |

Mutagenesis of the *sagA* gene in p*sagA* was accomplished using the Q5 Site-Directed Mutagenesis kit (NEB), according to the manufacturer’s instructions. The primers used for mutagenesis are reported in **Supplementary File 6**.
