# Supplementary material for: Secreted antigen A peptidoglycan hydrolase is essential for Enterococcus faecium cell separation and priming of immune checkpoint inhibitor therapy
Source: eLife. 2024 Jun 10;13:RP95297. doi: 10.7554/eLife.95297 (PMC11164530; doi:10.7554/eLife.95297)
Supplement: Supplementary file 6. [file elife-95297-supp6.docx]

**Supplementary File 6: Primers used in this study for SagA mutagenesis.**

| Mutation | Primer Name | Sequence (5’ to 3’) |
| --- | --- | --- |
| C431A | oSK479 | TCCAAGTGGATTTGACGCCTCAGGATTCACACGCTATGTTTACTTGCAAGTAACTGG |
|  | oSK480 | TCTTTACCGCCCCAAACATAAGGAGTACCAATATATTTG |
