# Supplementary material for: Secreted antigen A peptidoglycan hydrolase is essential for Enterococcus faecium cell separation and priming of immune checkpoint inhibitor therapy
Source: eLife. 2024 Jun 10;13:RP95297. doi: 10.7554/eLife.95297 (PMC11164530; doi:10.7554/eLife.95297)
Supplement: Supplementary file 7. [file elife-95297-supp7.docx]

**Supplementary File 7. Masses of peptidoglycan fragments were detected with MSD API-ES.**

| Peak^a^ | RT (min) | calculated  [M+H]^+^ | observed [M+H]^+^ | Proposed structure^b^ |
| --- | --- | --- | --- | --- |
| 1 | 4.96 | 826.4 | 828.6 | GM-tri |
| 2 | 6.46 | 698.2 | 698.4 | GMDP |
| 3 | 9.15 | 897.4 | 897.6 | GM-tetra |
| 4 | 11.85 | 940.4 | 940.6 | GM-tri (Asn)^c^ |
| 5 | 14.26 | 941.4 | 941.4 | GM-tri (Asp)^c^ |
| 6 | 16.95 | 1011.5 | 1011.6 | GM-tetra (Asn) |
| 7 | 18.70 | 1082.5 | 1082.6 | GM-penta (Asx) |
| 8 | 32.11 | 1819.9 | 1819.4 | 2GM-tri (Asx) - tetra |
| 9 | 33.77 | 1933.9 | 1934.4 | 2GM-tri (Asn) - tetra (Asn)^c^ |
| 10 | 35.69 | 1934.9 | 1935.2 | 2GM-tri (Asx) - tetra (Asx)^c^ |
| 11 | 38.15 | 2004.9 | 2005.2 | 2GM-tetra (Asn) - tetra (Asn)^c^ |
| 12 | 40.13 | 2006.9 | 2007.2 | 2GM-tetra (Asp) - tetra (Asp)^c^ |
| 13 | 46.52 | 2928.4 | 2929.2 | 3GM-tetra (Asx) - tetra (Asx) - tri (Asx)^c^ |
| 14 | 47.94 | 2929.4 | 2929.8 | 3GM-tetra (Asx) - tetra (Asx) - tri (Asx)^c^ |
| 15 | 51.29 | 3000.4 | 3000.6 | 3GM-tetra (Asx) - tetra (Asx) - tetra (Asx) |

^a.^ Peak numbers refer to Extended Data Figure 5a.

^b.^ GM, disaccharide (GlcNAc-MurNAc); 2GM, disaccharide-disaccharide (GlcNAc-MurNAc-GlcNAc-MurNAc); 3GM, disaccharide-disaccharide-disaccharide (GlcNAc-MurNAc-GlcNAc-MurNAc-GlcNAc-MurNAc); GM-Tri, disaccharide tripeptide (L-Ala-D-iGln-L-Lys); GM-Tetra, disaccharide tetrapeptide (L-Ala-D-iGln-L-Lys-D-Ala); GM-Penta, disaccharide pentapeptide (L-Ala-D-iGln-L-Lys-D-Ala -D-Ala).

^c.^ The assignment of the amide and the hydroxyl functions to either peptide stem is arbitrary.
